# D-MaPs - DNA-microarray projects: Web-based software for multi-platform microarray analysis

**DOI:** 10.1590/S1415-47572009000300030

**Published:** 2009-09-01

**Authors:** Marcelo F. Carazzolle, Taís S. Herig, Ana C. Deckmann, Gonçalo A. G. Pereira

**Affiliations:** Laboratório de Genômica e Expressão, Departamento de Genética e Evolução, Instituto de Biologia, Universidade Estadual de Campinas, Campinas, SPBrazil

**Keywords:** microarray, web service, software, affymetrix and nimblegen

## Abstract

The web application D-Maps provides a user-friendly interface to researchers performing studies based on microarrays. The program was developed to manage and process one- or two-color microarray data obtained from several platforms (currently, GeneTAC, ScanArray, CodeLink, NimbleGen and Affymetrix). Despite the availability of many algorithms and many software programs designed to perform microarray analysis on the internet, these usually require sophisticated knowledge of mathematics, statistics and computation. D-maps was developed to overcome the requirement of high performance computers or programming experience. D-Maps performs raw data processing, normalization and statistical analysis, allowing access to the analyzed data in text or graphical format. An original feature presented by D-Maps is GEO (Gene Expression Omnibus) submission format service. The D-MaPs application was already used for analysis of oligonucleotide microarrays and PCR-spotted arrays (one- and two-color, laser and light scanner). In conclusion, D-Maps is a valuable tool for microarray research community, especially in the case of groups without a bioinformatic core.

## Introduction

Biological approaches based on DNA microarrays are becoming instrumental to gain insights into gene expression from a genomic perspective. Since the technique supports the representation of the whole set of genes or genomic features on a single glass slide, virtually any cellular response can be investigated in detail. Moreover, microarray data analysis allows researchers to assess not only gene expression or regulation, but also gives support for characterization of gene functions and interactions.

However, despite the fact that microarrays are currently one of the most widespread techniques for functional studies in the fields of biology and medicine, it is extremely difficult to perform comparisons of the available data. First, differences in experimental design and microarray protocols must be considered when comparing gene expression data. The other problematic steps are mainly computational issues: data preprocessing, including data normalization, and statistical analysis for identification of differentially expressed genes. The preprocessing step is specific for each platform and aims to reduce technical artifacts with minor alterations to biological information. The statistical analysis summarizes the biological and technical replicates data by generating one value for the expression data (fold-change) and one value for the statistical result (p-value).

In an attempt to systematize analysis from the initial steps, by including raw data preprocessing, we describe here a web-based system - D-MaPs (DNA-Microarray Projects) - developed to manage and process microarray data obtained from different experimental designs (one- and two-color fluorescent labeling), equipment (light and laser scanners) and platforms (PCR-product spotted arrays and oligonucleotide arrays) (Schena *et al.*, 1995; Nuwaysir *et al.* 2002). The system has been implemented to manage GeneTAC, ScanArray, CodeLink, NimbleGen and Affymetrix microarray platforms.

D-MaPs features a simple interface to allow researchers unfamiliar with statistics and graphical development to easily obtain their results. The user can access the system using the version installed on a server located in our bioinformatics laboratory. D-Maps system also provides the output data in GEO (Gene Expression Omnibus) submission format, commonly requested by many periodicals for articles based on microarray data.

## Methods

The D-MaPs application was written in Perl and R computer languages, supports entry of various file formats and contains implementations of diverse Bioconductor packages (Gentleman *et al.*, 2004) for normalization and statistical analysis. It uses standard open source modules such as CGI.pm and DBI.pm. The system requires the Linux operating system with a Web server (*e.g.*, Apache) and MySQL server installed. The system runs on a high performance server with large amount of memory (16 Gb of ram). The main advantage of this architecture is that the server performs the majority of the processing work, which makes it unnecessary for external users to have high performance computers.

With exception of the database management and dynamic HTML pages generation, all calculations are performed in R functions of the Bioconductor packages including graphics generation. The R scripts are dynamically generated while the user navigates on the interface.

## Program Description

**Figure 1:**
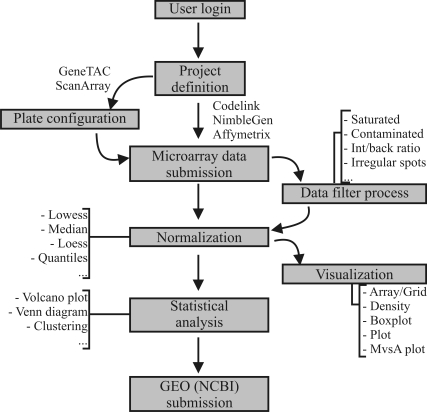
The D-maps analysis workflow. The web interface guides the users through these steps.

For each user D-maps program provides a separate workspace, called projects, for storing and analyzing microarray data. The web interface guides the user through further steps, such as project definition, upload data, data preprocessing and statistical analysis, necessary to perform the analysis of microarray data. During each step the user has access of all analyzed data in text or graphical format. Figure 1 shows the main features of the system.

###  Project description and data submission

**Figure 2:**
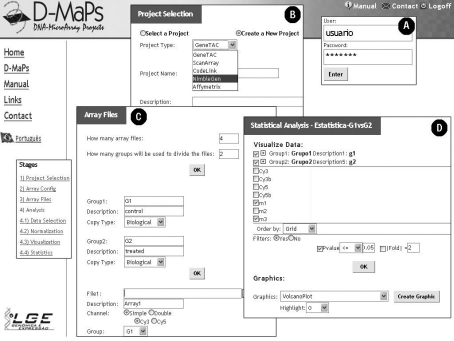
Some D-maps Web interfaces: (A) User and password control; (B) Project description; (C) Array details and upload data - in this step the user defines the groups and provides details about each array file (One or two channel, dye swap and experimental replicates); (D) Statistical analysis - the user defines the threshold values for fold-change and p-value to obtain the differential expressed genes.

First of all, the user creates a login/password (Figure 2A). Then, he is prompted to create a project and provide some details about it, including type of scanner (platform) used for image acquisition (selection of 1 of 5 platforms currently supported by D-Maps: GeneTAC, ScanArray, CodeLink, NimbleGen and Affymetrix) and a basic description. The project definition interface is shown in Figure 2B.

The next stage is designed for the submission of raw data files (Figure 2C). The files can be divided into groups according the experimental design (*e.g.*, one group is formed by files representing data from experimental samples and other group from control samples). In the case of projects based on GeneTAC or ScanArray platforms, it is also necessary to define whether microarrays are one- or two-color (*i.e.*, single or dual channel images), and if the file represents the dye-swap replicates or not. For Affymetrix, Codelink and NimbleGen platforms, the raw data files are defined as single channel. Also, the file formats are different according to the platform used: CSV file for GeneTac and ScanArray platforms, TXT file for Codelink, XYS and NDF files for NimbleGen and CEL files for Affymetrix.

###  Data preprocessing

**Figure 3:**
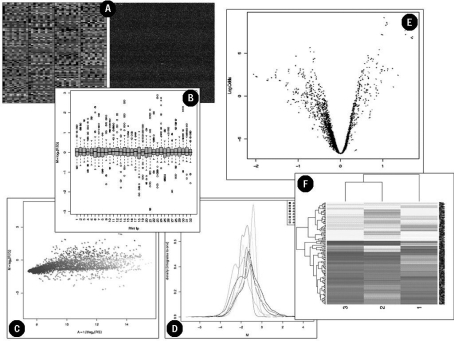
Graphics visualizing some steps during the analysis workflow: (A) spatial plot showing the spot intensities; (B) Box plot showing the grid variation in one array; (C) M-A plot summarizing the spot intensities in a two channel experiment; (D) Density plot summarizing the distribution of intensities between arrays; (E) Volcano plot showing the differential expressed genes; (E) Cluster analysis grouping the genes with similar expression pattern.

Data preprocessing is an essential step in the analysis of microarray data. Basically, it consists of filtering the data using scanner flag information, background correction, normalization intra- and between-slides calculations. The difficulty in this step is that raw data generated by each platform is flagged by a specific set of identifiers according methods and parameters that are platform specific. D-maps can perform this step using the best set of methods and parameters for each platform, but the user can change this information depending on particularities of the data. The graphical tools are important to resume the information contained in the microarray data during the preprocessing steps. Figure 3 shows some graphics displayed by D-Maps.

*Flag determination*. After submission of raw data files, an automatic (adjustable) assignment is made based on the quality of intensity signals (saturated, contaminated, irregular), excluding artifactual data prior to further analysis. As mentioned above, the flags are platform dependent.

*Background correction*. Usually, the spot intensities are corrected for background intensities by subtraction of the latter. For the Affymetrix platform, the specific background subtraction methods are available to users: RMA and MAS algorithms (Eschrich and Hoerter, 2007). For the Codelink platform, the Subtract method (Diez *et al.*, 2007) is implemented.

*Data normalization*. A large number of normalization tools are available in this step for one- or two-color microarrays. All normalization methodologies perform the log transformation (base 2) of fluorescence intensities prior to the analysis. In the case of two-color microarrays (available for GeneTac and ScanArray platform), the log ratio of intensities (Log2 Cy5/Cy3) is calculated.

*Two-color microarrays.* For two-color experiments the system performs both intra- and between-slides calculations (Irizarry *et al.*, 2003; Yang *et al.*, 2002). The intra-slide normalization process is necessary to remove such specific variations, *e.g.* differences in labeling efficiency of the two fluorescent dyes. D-Maps allows the user to choose among Lowess-scaled print-tip (Yang *et al.*, 2002), Lowess print-tip (Yang *et al.*, 2002), Global Lowess (Yang *et al.*, 2002) and Median (Quackenbush, 2002) methods to perform the intra-slide normalization. After that, all normalized log-ratios will be centered around zero. Since distinct slides present non-homogeneous variations in their log-ratio values (the so-called “scale effect”), it must be corrected using between-slides normalization methods. D-maps performs between-slides normalization using Quantile method (Bengtsson and Bengtsson, 2006).

*One-color microarrays*. In the case of Codelink platform, just the Cyclic Lowess method is implemented (Wu *et al.*, 2005; Diez *et al.*, 2007) for data normalization. For Nimblegen platform, the Quantile normalization is implemented. Finally, for the Affymetrix Genechip platform, the normalization methods were implemented using RMA and MAS algorithms (loess, constant, contrasts, invariantSet, qspline, quantiles and quantiles.robust).

###  Statistical analysis applied to detection of differentially expressed genes

After normalized, data is statistically analyzed both for reproducibility of replicated values and for significance of the differences among conditions to be compared. To perform this step, D-Maps has implemented the Limma package (Smyth, 2004) from Bioconductor. Limma uses linear models to analyze the microarray experiments (eBayes-moderated t-test). The statistical analysis summarizes the biological and technical replicates data by generating two values for each probe: (i) fold-change value: summarizes the intensity of each gene by calculating the average of normalized values in each experimental replicate; (ii) p-value: summarizes the coherence between the normalized values in each experimental replicate. Figure 2D shows the statistical analysis interface, in which the user is prompted to define the threshold values for fold-change and p-value. This set of parameters generates the set of differentially expressed genes, which are displayed graphically (Volcano and clustering plot, shown in Figure 3E and 3F, respectively) and in plain text format (txt files).

## Results and Discussion

In recent years, Bioconductor has become the reference tool for the analysis of microarray data because it is based on up-to-date algorithms. However, for scientists without programming experience, the command line usage of Bioconductor is too difficult. Therefore, many analysis tools with a graphical user interface and powerful computing servers have been developed, including web-based tools like Webarray (Xia *et al.*, 2005), MIDAW (Romualdi *et al.*, 2005), MAGMA (Rehrauer *et al.*, 2007) or CarmaWEB (Rainer *et al.*, 2006).

In most cases, however, there are limitations concerning platforms and type of data formats supported by these software programs. For instance, data from two-color microarrays requires the external manipulation before the input step. Since the raw data files derived from the image analysis software are usually large and difficult to handle, especially for inexperienced users, researchers working with two-color microarray data normally have to navigate several websites and transfer the data between the servers to complete their analysis. With this in mind, D-Maps software was designed and implemented. Table 1 presents a comparative view of the main features of Webarray, MIDAW, MAGMA, CarmaWEB and D-Maps software programs.

An original feature presented by D-Maps software is GEO (Gene Expression Omnibus) submission format service. Nowadays, scientific articles based on microarray data must be accompanied by submission of both raw and processed data to public databases, as NCBI/GEO Database (Barrett *et al.*, 2007). This process requires data to follow some format standards, including experimental design and technical requirements of platforms. D-Maps prompts users to give all necessary information during raw data submission, therefore output files containing GEO formats are automatically generated.

The D-MaPs application has already been used for analysis of oligonucleotide microarrays (one-color, laser scanner) (Deckmann AC, Rocco SA, Marin RM, Rolim Filho LA, Carazzolle MF, Barau JG, Pereira GAG and Franchini KG, unpublished data) and PCR-spotted arrays (one- and two-color, laser and light scanner) (Rincones *et al.*, 2008; Rubin MS, Deckmann MF, Carazzolle MF, Parizzi LP, Pereira GAG and Annichino-Bizzacchi JM, unpublished data; Lepikson-Neto J, Deckmann AC, Carazzolle MF and Pereira GAG, unpublished data). In the first study, D-Maps performed the pre-processing of raw data obtained from Codelink bioarrays according Codelink package of Bioconductor repository: inter-slide normalization by Lowess method and differential expression by eBayes-moderated t-test for microarrays. In later studies, raw data obtained from distinct scanners were analyzed by D-Maps according to the Aroma and Limma package of Bioconductor repository: intra-slide normalization (Lowess or median), inter-slide normalization (Quantile) and differential expression by eBayes-moderated t-test for microarrays. Other studies are currently in progress, for which GEO submission support service has been implemented.

## Conclusions

In this study we describe a web-based system, D-MaPs (DNA-Microarray Projects), developed to manage and process microarray data obtained from distinct experimental designs (one- and two-color fluorescent labeling) and platforms (GeneTAC, ScanArray, CodeLink, NimbleGen and Affymetrix). D-MaPs features a simple interface to allow researchers who are unfamiliar with computation and statistics to easily obtain results of differential gene expression in graphical and plain text formats. The D-Maps system also provides output data in GEO (Gene Expression Omnibus) submission format. In conclusion, D-Maps is a valuable tool for microarray research community, especially in the case of groups without a bioinformatic core.

## Figures and Tables

**Table 1:** Some surveyed systems and their characteristics.

Characteristics	WebArray	MIDAW	MAGMA	CarmaWeb	D-maps
Web interface	X	X	X	X	X
User and project management	X				X
One channel microarray data	X			X	X
Two channel microarray data	X	X	X	X	X
Preprocessing data analysis	X	X	X	X	X
Detection of differentially expressed	X	X	X	X	X
Visualization tools	X	X	X	X	X
Cluster analysis		X		X	X
Submission at GEO database (NCBI)					X
Microrray platforms supported:					
Affymetrix	X			X	X
Codelink			X		X
GeneTac		X		X	X
Nimblegen					X
ScanArray		X		X	X
